# Exercise After Coronary Stenting: Potential Effects on in-Stent Restenosis

**DOI:** 10.1007/s11883-026-01455-7

**Published:** 2026-08-19

**Authors:** Luigi Spadafora, Stefano Cacciatore, Monica Salvi, Federico Russo, Lorenzo Lo Sasso, Attilio Lauretti, Francesco Perone, Nicola Pierucci, Pierre Sabouret, Giorgio Zangari, Alfredo De Vito, Giuseppe Biondi Zoccai, Marco Bernardi

**Affiliations:** 1 Division of Cardiology and Interventional Cardiology, Santa Maria Goretti Hospital, Latina, 04100 Italy; 2 Department of Medical-Surgical Sciences and Biotechnologies, Sapienza University of Rome, Latina, Italy; 3 Department of Physiology and Aging, College of Medicine, University of Florida, Gainesville, FL USA; 4 School of Medicine, College of Medicine and Health, University College Cork, Cork, Ireland; 5 Department of Medical and Surgical Sciences (DIMEC), Alma Mater Studiorum, University of Bologna, Bologna, Italy; 6 Division of Cardiology, Sant’Andrea University Hospital, Rome, 00189 Italy; 7 Catholic University of the Sacred Heart, Rome, Italy; 8 Cardiac Rehabilitation Unit, Rehabilitation Clinic “Villa delle Magnolie”, Castel Morrone, Caserta, 81020 Italy; 9 Department of Cardiovascular, Respiratory, Nephrological, Anesthesiological and Geriatric Sciences “Sapienza”, University of Rome, Rome, 00161 Italy; 10 Cardiology Department, Institut de Cardiologie, Centre Hospitalier Universitaire La Pitié-Salpêtrière, Sorbonne Université, Paris, France; 11 Sapienza University of Rome, Rome, Italy; 12 Maria Cecilia Hospital, GVM Care and Research, Cotignola, 48033 Italy

**Keywords:** In-stent restenosis, Exercise training, Cardiac rehabilitation, Percutaneous coronary intervention, Endothelial function, Drug-eluting stent

## Abstract

**Purpose of Review:**

Structured exercise is a cornerstone of secondary prevention after percutaneous coronary intervention (PCI), yet its specific relationship with in-stent restenosis (ISR) is less well characterized. This narrative review summarizes the biological rationale, clinical evidence, safety, and practical prescription of exercise training in patients with coronary stents, with a focus on its potential impact on ISR.

**Recent Findings:**

Exercise training improves endothelial nitric oxide bioavailability, reduces systemic inflammation, and favorably modulates neointimal proliferation in experimental and clinical studies. Pooled clinical data suggest that exercise-based cardiac rehabilitation after PCI is associated with a reduction in angiographically defined restenosis, although individual trials, largely from the bare-metal and early drug-eluting stent (DES) eras, have shown inconsistent results on binary restenosis endpoints. Available contemporary data do not suggest an excess risk of stent thrombosis, clinically relevant arrhythmias, or adverse ventricular remodeling when exercise is initiated in clinically stable patients using an individualized and supervised approach.

**Summary:**

The direct anti-restenotic effect of exercise remains biologically plausible and clinically suggestive but is not yet firmly established, particularly in the contemporary thin-strut DES era. Nevertheless, the well-documented benefits of exercise on functional capacity, endothelial health, and cardiovascular prognosis justify its role as an essential, time-sensitive component of post-PCI care. Larger, contemporary trials with standardized intracoronary imaging endpoints are needed to clarify whether, and in which patient subsets, exercise meaningfully affects the biology of ISR.

## Introduction

In-stent restenosis (ISR) remains the most common cause of stent failure after percutaneous coronary intervention (PCI), despite the widespread adoption of drug-eluting stents (DES) [1, 2]. ISR is conventionally defined as a diameter stenosis of at least 50% within the stented segment or within 5 mm of its edges [1]. PCI for ISR still accounts for approximately 5–10% of all PCI procedures [1]. With contemporary DES, ISR continues to accrue at an estimated annual rate of 1–2% during long-term follow-up [3, 4]. More recently, a meta-analysis reported a pooled incidence of DES-ISR of approximately 13% across contemporary cohorts [5]. Compared with PCI for de novo lesions, ISR is associated with a higher likelihood of recurrent symptoms, repeat revascularization, and, not infrequently, presentation as an acute coronary syndrome [2, 3]. The pathophysiology of ISR has evolved from a purely mechanical, device-centered paradigm to a more integrated model that recognizes ISR as the product of interacting patient-, lesion-, procedure-, and stent-related factors, including diabetes mellitus, stent length and number, lesion length, involvement of the left anterior descending artery, and prior PCI, whereas a higher left ventricular ejection fraction appears protective [1, 2, 5]. Early ISR is predominantly driven by endothelial denudation, local inflammation, oxidative stress, and neointimal hyperplasia, whereas later phases are increasingly characterized by vascular smooth muscle cell phenotypic switching and in-stent neoatherosclerosis, a substrate that may itself confer plaque instability and thrombotic risk and is best characterized with optical coherence tomography [2–4, 6]. Contemporary angiographic and imaging-based classification systems, such as the Mehran and Waksman classifications, further separate ISR according to its mechanical, biological, or mixed underlying substrate, informing individualized management [2]. This shared biological ground, endothelial dysfunction, inflammation, and maladaptive vascular repair, is precisely the terrain on which structured exercise is known to act favorably in other cardiovascular contexts, providing the mechanistic rationale for its investigation as a non-pharmacological, adjunctive strategy after stenting.

Exercise-based cardiac rehabilitation is already a guideline-endorsed cornerstone of secondary prevention after PCI, with well-established benefits on functional capacity, anginal symptoms, cardiometabolic risk, and cardiovascular mortality [7, 8]. Whether these benefits extend to a measurable reduction in the risk of ISR specifically, however, is a narrower and less settled question, complicated by the heterogeneity of available studies, the evolution of stent technology across the study period, and the practical difficulty of imaging the stented segment serially in clinical trials. The present narrative review addresses four complementary questions: (i) what is the biological basis linking exercise training to vascular healing after stenting; (ii) what does the clinical trial and meta-analytic evidence show regarding exercise and ISR; (iii) is early exercise after coronary stenting safe, and how should it be timed; and (iv) how should exercise intensity and monitoring be practically approached in this population.

## Literature Search and Review Approach

This narrative review was based on literature searches conducted in PubMed/MEDLINE, Scopus, and Web of Science up to June 2026. Search terms included combinations of “in-stent restenosis”, “coronary stent”, “drug-eluting stent”, “percutaneous coronary intervention”, “exercise”, “exercise training”, “physical activity”, “cardiac rehabilitation”, “vascular healing”, “endothelial function”, “neointimal hyperplasia”, “exercise safety”, and “exercise prescription”. The reference lists of relevant reviews and original studies were also screened to identify additional publications. The evidence was organized around four predefined domains: biological mechanisms linking exercise to vascular healing, restenosis-related clinical outcomes, safety and timing of exercise initiation, and practical exercise prescription after PCI. Priority was given to clinical practice guidelines, randomized and controlled studies, systematic reviews, meta-analyses, and contemporary observational studies directly relevant to PCI and coronary stenting. Evidence directly assessing ISR was considered separately from studies reporting surrogate vascular endpoints or broader clinical outcomes after PCI.

## Biological Mechanisms Linking Exercise to Vascular Healing After Stenting

Coronary stent implantation causes endothelial injury and triggers inflammation and activation of platelet and coagulation pathways. Vascular healing and endothelial recovery evolve over the first weeks to months after stent implantation, with substantial variability according to procedural, device-related, and patient-related characteristics [9, 10]. Against this background, regular exercise is biologically plausible as a protective intervention. However, much of the available clinical mechanistic evidence is indirect and derives from patients with coronary artery disease rather than specifically from populations undergoing coronary stenting. Hambrecht and colleagues showed, in patients with stable coronary artery disease, that exercise training increased phosphorylation of endothelial nitric oxide synthase (eNOS) and improved endothelium-dependent vasodilation, findings of potential relevance to the post-stenting milieu [11]. More direct evidence comes from experimental models of vascular intervention: physical training has been shown to increase vascular eNOS expression and activity and to reduce neointimal proliferation after balloon angioplasty and arterial stenting in animal models [12]. Additional supportive, although not stent-specific, evidence comes from carotid injury models, in which localized nitric oxide (NO) delivery inhibited neointima formation [13]. Collectively, these findings support the broader biological premise that NO exerts pleiotropic vascular effects, promoting vasodilation while inhibiting platelet activation, smooth muscle cell proliferation, and neointimal growth [12, 14].

Beyond the NO pathway, exercise training modulates several inflammatory pathways that drive vascular healing after PCI. Here again, most clinical evidence is extrapolated from broader coronary populations. Regular exercise training has been associated with lower C-reactive protein levels and a more favorable circulating cytokine profile in patients with coronary heart disease [15], while improvements in endothelial function have been demonstrated in complementary clinical studies [11]. More broadly, exercise-induced reductions in circulating pro-inflammatory cytokines, together with increases in shear and circumferential stress, may promote structural remodeling and functional improvement of the vessel wall [9, 16]. Stent-specific clinical evidence remains limited. In a small randomized study comparing aerobic interval training with moderate continuous training after DES implantation, plaque burden and necrotic core content decreased at the distal stent edges in both groups, with larger reductions after interval training; however, the absence of a non-exercise control group limits causal interpretation [17].

**Fig. 1:**
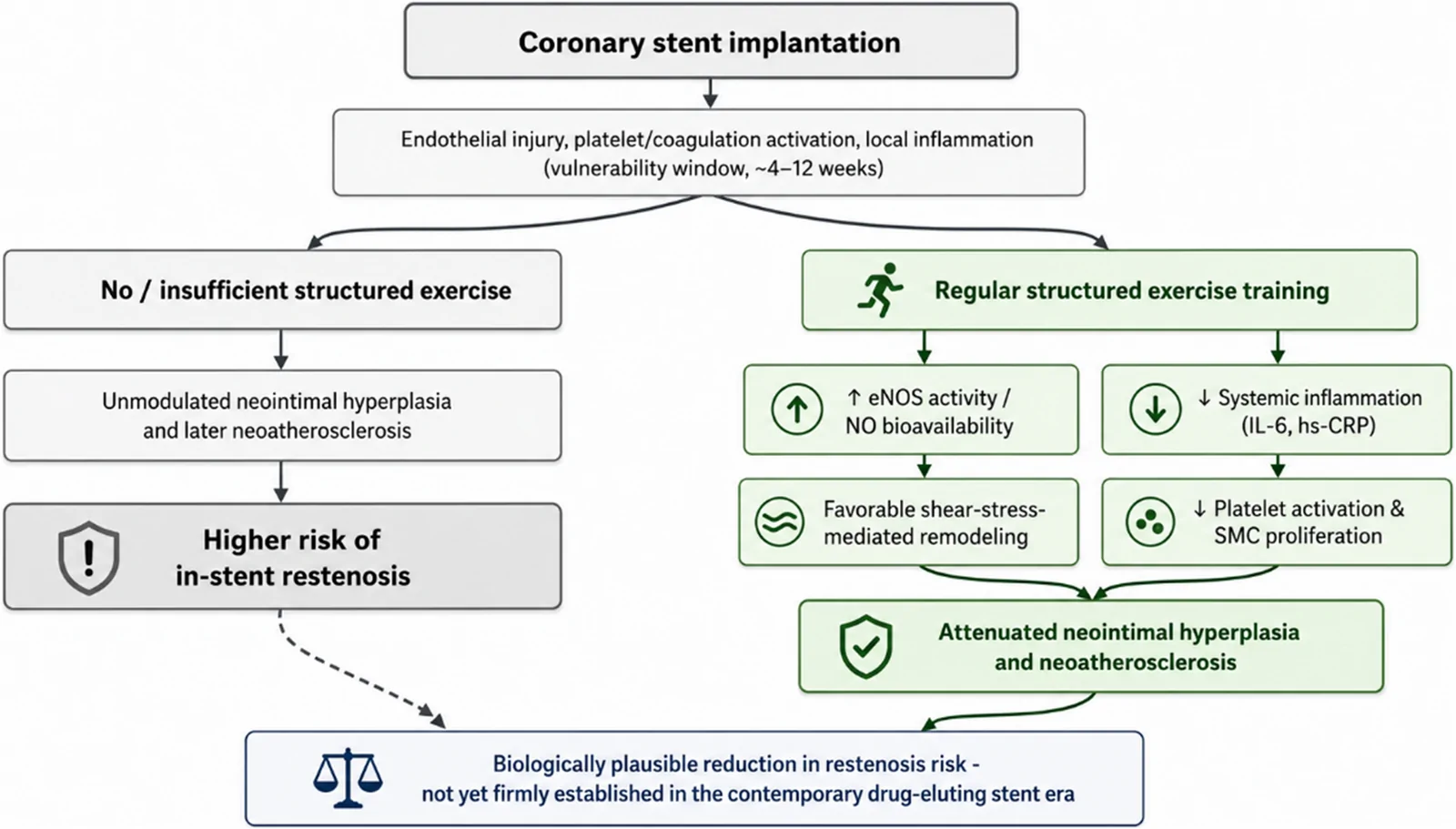
Biological mechanisms linking exercise training to vascular healing after coronary stenting and their putative relationship with in-stent restenosis. Abbreviations: ISR, in-stent restenosis; eNOS, endothelial nitric oxide synthase; NO, nitric oxide; IL-6, interleukin-6; hs-CRP, high-sensitivity C-reactive protein; SMC, smooth muscle cell

Taken together, the mechanistic framework linking exercise to vascular healing after stenting is based on a combination of direct evidence from experimental stent models and limited post-DES clinical studies, together with indirect evidence extrapolated from patients with coronary artery disease and other models of vascular injury. These data support a coherent, multi-pathway model in which exercise training improves endothelial function and NO bioavailability, attenuates oxidative stress and systemic inflammation, and modulates vascular smooth muscle cell proliferation, each of which intersects directly with the mechanisms implicated in both early neointimal hyperplasia and later neoatherosclerotic ISR [3, 4, 11, 12]. Figure 1 summarizes this multi-pathway model, contrasting the unmodulated healing response with the vasculoprotective adaptations induced by regular structured exercise. Thus, the biological rationale is well supported, whereas direct evidence that exercise modifies the development of ISR, particularly after contemporary DES implantation, remains limited.

## Exercise Dose, Adherence, and Guideline Recommendations

Aerobic physical activity is a cornerstone of cardiovascular prevention. The 2021 ESC guidelines recommend at least 150 min/week of moderate-intensity physical activity, or 75 min/week of vigorous-intensity activity, with additional benefit accruing as activity volume increases; even amounts below this threshold are better than none, and exercise training more generally reduces all-cause and cardiovascular mortality [7, 18, 19]. These recommendations provide a pragmatic framework for exercise prescription after PCI, although no specific exercise dose has been validated for the prevention of ISR. Sustained participation in cardiac rehabilitation is associated with a durable survival benefit: in one large cohort, attendance at cardiac rehabilitation was associated with lower all-cause mortality at 14 years of follow-up [20]. This observation supports the importance of continued participation rather than short-term program enrollment alone. Despite this evidence base, adherence remains a major limitation in practice. A substantial proportion of patients, at least 50% in some series, fail to achieve recommended activity levels after PCI, with correspondingly modest self-reported physical capacity at one-year follow-up [21, 22]. This gap between the prescribed and the effectively achieved exercise dose is particularly relevant when interpreting studies of restenosis, in which adherence is often incompletely reported or assessed only indirectly. Exercise-based interventions have been shown to improve cardiac function, functional capacity, and cardiovascular outcomes after PCI and pooled analyses suggest a possible reduction in restenosis [23–26]. However, evidence linking a specific exercise volume, training duration, or level of adherence to the risk of ISR remains limited [27–31].

Strategies aimed at improving long-term adherence may therefore be clinically relevant, even though their direct effect on restenosis has not been established. A 2026 randomized controlled trial of a persuasive, app-based eHealth cardiac rehabilitation platform added to standard care improved physical activity levels, exercise endurance, and exercise self-efficacy after PCI compared with standard rehabilitation alone, illustrating a growing role for digitally supported programs in sustaining exercise dose over time [27–31]. Accordingly, current exercise prescription after PCI should remain based on guideline-recommended activity targets, individualized progression, and strategies supporting sustained participation, rather than on a specific anti-restenotic exercise threshold.

## Clinical Evidence: Exercise Training and In-Stent Restenosis

Clinical data directly linking exercise training to a reduction in ISR are less abundant than the mechanistic rationale would suggest. Table 1 summarizes the design and restenosis-related findings of the principal studies discussed below. Early evidence comes from the ETICA trial, in which Belardinelli and colleagues randomized 118 patients after successful coronary angioplasty, with or without stenting, to a supervised 6-month exercise program or usual care [32]. Exercise training produced a significant 26% increase in peak oxygen consumption (*p* < 0.001) and a 20.3%-point lower composite event rate during follow-up (11.9% vs. 32.2%; RR 0.71, 95% CI 0.60–0.91, *p* = 0.008); however, no significant difference was observed in binary restenosis rates at follow-up (29% vs. 33%; P = NS), even though trained patients showed a lower degree of residual diameter stenosis, suggesting a possible favorable influence on the extent of neointimal proliferation that did not translate into a difference on a dichotomous angiographic endpoint [32]. An additional aspect worth considering when interpreting ETICA is its background medical therapy: lipid-lowering drugs were not permitted during the study, a design feature that reflects the fact that, at the time of enrollment (1993), the role of statins in secondary prevention had not yet been established.

**Table 1:** Key studies evaluating exercise training and restenosis-related endpoints after percutaneous coronary intervention

Study (year)	Design and population	Intervention	Key restenosis-related findings
Belardinelli et al., ETICA trial (2001)	RCT; 118 patients after coronary angioplasty, with or without stenting	6-month supervised exercise vs. usual care	No difference in binary restenosis (29% vs. 33%, P = NS); lower residual diameter stenosis with training (*p* = 0.045)
Munk et al. (2009)	RCT; patients after PCI with stent implantation	6-month high-intensity interval training vs. control	Smaller late luminal loss (0.10 vs. 0.39 mm, *p* = 0.01); improved endothelial function; lower hs-CRP
Kim et al. (2012)	Cohort; patients receiving different DES generations	Exercise-based cardiac rehabilitation	Lower late luminal loss with rehabilitation (0.16 vs. 0.39 mm), consistent across three DES generations
Lee et al. (2013)	Controlled cohort study; 74 patients with acute myocardial infarction treated with DES	Exercise training vs. control	Lower late luminal loss at 9 months (0.14 vs. 0.54 mm, *p* = 0.02)
Fu et al. (2019)	Systematic review and meta-analysis of RCTs	Exercise-based cardiac rehabilitation after PCI	Lower restenosis (OR 0.46, 95% CI 0.26–0.82) and smaller late luminal loss (mean difference − 0.33 mm) with exercise
Zhang and Chang (2019)	Meta-analysis; 10 RCTs, 1,274 patients	Exercise after PCI vs. control	Angiographic restenosis risk ratio 0.36 (95% CI 0.16–0.83
Li et al. (2024)	Meta-analysis of RCTs	Exercise-based cardiac rehabilitation after PCI	Lower coronary restenosis (RR 0.10, 95% CI 0.01–0.76; based on 2 of 13 pooled trials)

*RCT* randomized controlled trial, *DES* drug-eluting stent, *VO2* oxygen consumption/uptake, *MACE* major adverse cardiovascular events, *hs-CRP* high-sensitivity C-reactive protein, *LVEF* left ventricular ejection fraction, *CI* confidence interval, *RR* risk ratio, *OR* odds ratio, *HDL-C* high-density lipoprotein cholesterol, *ISR* in-stent restenosis

Subsequent controlled studies have generally pointed in a favorable direction. In a small randomized trial, Munk and colleagues reported that six months of high-intensity interval training after PCI with stent implantation reduced late luminal loss and was accompanied by improved endothelial function and lower systemic inflammation [33]. In a controlled cohort study of patients undergoing PCI with DES for acute myocardial infarction, Lee and colleagues observed a similar pattern: at 9 months, late luminal loss was significantly lower in the exercise group (0.14 ± 0.57 mm vs. 0.54 ± 0.88 mm; *p* = 0.02), an effect associated with a significantly greater increase in exercise capacity (VO2max, *p* < 0.001) and HDL cholesterol (*p* = 0.03) [34]. Kim and colleagues further reported an association between participation in exercise-based cardiac rehabilitation and lower late luminal loss across different generations of DES [35] Table 2.

**Table 2:** Guideline-supported principles summarize recommendations from international cardiovascular prevention, sports cardiology, and cardiac rehabilitation guidance [7, 38, 41]. Practical applications represent the authors’ interpretation of these principles according to common post-PCI clinical scenarios and should not be considered universally validated timing thresholds. Exercise initiation and progression should be individualized according to clinical stability, vascular access-site healing, residual ischemia, ventricular function, arrhythmic risk, functional capacity, and comorbidities

Clinical scenario	Guideline-supported principle	Practical application proposed in this review	Suggested exercise approach	Monitoring and precautions
Uncomplicated PCI, immediate post-procedural phase	Early mobilization is appropriate after clinical stabilization, with precautions related to vascular access-site recovery [7, 38]	Begin light ambulation after clinical and access-site assessment	Low-intensity walking, progressively increased according to symptoms and functional tolerance	Avoid heavy lifting and excessive strain involving the access limb until adequate healing
Elective PCI for chronic coronary syndrome	Early enrollment in cardiac rehabilitation and individualized exercise prescription are recommended in clinically stable patients [7, 38]	Structured exercise may generally begin within the first days to weeks, provided there is no uncontrolled residual ischemia or relevant procedural complication	Moderate-intensity continuous aerobic training; resistance exercise introduced after initial aerobic conditioning	Consider closer supervision in patients with reduced LVEF, significant residual disease, inducible ischemia, or limited functional capacity
Non-ST-elevation acute coronary syndrome treated with PCI	Cardiac rehabilitation should begin early after stabilization and should include formal risk stratification [7, 38, 41]	Initiate structured aerobic exercise during early recovery or shortly after discharge, according to clinical course	Moderate-intensity training with gradual progression; resistance training introduced later according to individual status	Assess ischemia, ventricular function, arrhythmic burden, symptoms, and exercise capacity
Uncomplicated ST-elevation myocardial infarction treated with PCI	Early, individualized cardiac rehabilitation is recommended once clinical stability has been achieved [7, 38]	Begin during early recovery, preferably within a supervised program	Supervised moderate-intensity aerobic training; higher-intensity protocols may be considered in selected, clinically stable patients	Greater supervision is advisable during early recovery and before progression to higher intensities
High-risk or clinically complex patients	Exercise prescription and monitoring should be guided by formal risk stratification, including ventricular function, ischemia, arrhythmias, symptoms, and functional capacity [41]	Rehabilitation should not necessarily be delayed, but initiation and progression should be closely supervised and individualized	Lower initial intensity, shorter sessions if needed, and gradual progression in duration before intensity	Cardiology-led assessment at baseline, with reassessment according to clinical status; consider ECG, imaging, exercise testing, functional capacity, and comorbidities

*ECG* electrocardiography, *LVEF* left ventricular ejection fraction, *PCI* percutaneous coronary intervention, *ST* ST segment

At the pooled level, two systematic reviews and meta-analyses, by Fu et al. and by Li et al., have confirmed a reduction in restenosis risk with exercise-based rehabilitation after PCI [36, 37], with Fu et al. additionally reporting smaller late luminal loss with exercise (mean difference − 0.33 mm) [36]. In a meta-analysis by Zhang and Chang including 10 randomized controlled trials and 1,274 patients, regular exercise after PCI was associated with a significant reduction in angiographically defined restenosis (risk ratio 0.36; 95% CI 0.16–0.83), corresponding to an approximate 64% relative risk reduction, alongside an improvement in left ventricular ejection fraction (mean difference 2.82; 95% CI 1.50–4.14) [26]. Interpretation of this pooled estimate requires caution, however, as most included studies were conducted in the bare-metal stent or early-generation DES era; the external validity of these results to contemporary practice, dominated by newer-generation thin-strut DES and a different biological substrate of ISR, therefore remains uncertain.

Important limitations temper these overall favorable signals. Available studies are heterogeneous, frequently underpowered for angiographic endpoints, and provide limited insight into subgroup-specific effects, even though ISR risk is strongly influenced by patient- and lesion-related variables such as diabetes mellitus, lesion complexity, stent length, and vessel diameter, factors independently confirmed in a 2024 pooled analysis of risk factors for DES-ISR [5]. Diabetes, in particular, is associated with diffuse restenosis patterns, enhanced neointimal proliferation, and increased target lesion failure; although exercise improves glycemic control and several cardiometabolic parameters, whether it can meaningfully offset the biological burden of diabetic ISR remains uncertain. Similarly, longer stented segments are exposed to greater inflammatory and proliferative stimuli, and it is unclear whether exercise can mitigate these effects in a clinically meaningful way.

Overall, while the direct effect of exercise training on ISR remains incompletely defined, the clinical relevance of structured exercise after PCI extends well beyond this single endpoint. The consistent and well-established benefits of exercise on functional capacity, endothelial function, cardiometabolic profile, and cardiovascular outcomes support its role as a cornerstone of secondary prevention, irrespective of a measurable impact on restenosis [8, 36, 37].

## Safety and Timing of Exercise After Coronary Stenting

### Clinical Relevance and Knowledge Gap

Structured exercise and cardiac rehabilitation are strongly recommended by international societies such as the American Heart Association (AHA) and the ESC [7, 38], Historical concerns have focused on the transient biological vulnerability of the recently stented segment and on a theoretical increase in ischemic or thrombotic risk during early exercise. In clinical practice, however, the timing of rehabilitation is influenced not only by stent healing but also by clinical stability, residual ischemia, ventricular function, arrhythmic risk, and vascular access-site recovery.

### Pathophysiological Rationale: the Vulnerability Window

Coronary stenting causes endothelial injury and activates platelet and coagulation pathways. Vascular healing and endothelial recovery evolve over the first weeks to months, with substantial variability across patients and stent platforms [9, 10]. During the early post-PCI period, acute high-intensity exercise may transiently increase sympathetic activity, inflammation, and platelet activation, providing a theoretical rationale for caution during the early post-PCI period. This acute response should, however, be distinguished from the effects of regular exercise training, which improves endothelial function, increases NO bioavailability, and promotes a more favorable inflammatory profile [11, 12]. The available biological evidence therefore supports progressive and individualized exercise initiation rather than the routine postponement of cardiac rehabilitation after an uncomplicated procedure.

### Timing of Exercise Initiation

After uncomplicated PCI, early mobilization and light ambulation are generally appropriate, while heavy lifting and excessive strain on the vascular access limb should be avoided until access-site healing is adequate. Cardiac rehabilitation is best conceptualized as a continuum of care, beginning in-hospital (phase I), transitioning to structured outpatient or residential programs shortly after discharge (phase II), and continuing lifelong (phase III); contemporary ESC and AHA guidance favors early enrollment in comprehensive rehabilitation after both acute and chronic coronary syndromes, tailored to age, frailty, comorbidities, and risk [7, 38]. This is reinforced by a meta-analysis by Haykowsky and colleagues, which demonstrated that prompt initiation of exercise training after myocardial infarction provides the greatest benefit on ventricular remodeling, with each week of delay requiring a progressively longer period of training to achieve comparable benefit [39].

Timing should be individualized according to the clinical scenario. In patients undergoing elective PCI for chronic coronary syndromes, structured exercise can generally begin within the first days to weeks, provided there is clinical stability, appropriate access-site healing, and no uncontrolled residual ischemia. High-risk features such as a left ventricular ejection fraction below 50%, severe residual stenoses, or inducible ischemia do not contraindicate rehabilitation but warrant closer supervision and more gradual progression. In patients with acute coronary syndromes treated with PCI, a similarly proactive but more closely supervised approach is appropriate: aerobic training may be initiated during early recovery once the patient is clinically stable, while resistance training should be introduced later according to functional status, access-site healing, ischemic burden, and arrhythmic risk. A recent meta-analysis of 16 studies and 1,810 patients found that the timing of cardiac rehabilitation initiation after PCI for acute myocardial infarction within the first month had no significant effect on arrhythmia, coronary restenosis, angina, or measures of left ventricular remodeling and functional capacity, while early training, center- or home-based, was associated with improved ventricular function, exercise capacity, and low rates of adverse events [40]. These findings are reassuring, although heterogeneity among the included programs and the low frequency of serious adverse events limit the ability to define a single optimal starting point or to exclude small differences in risk.

### Exercise Prescription and Program Duration

Risk stratification prior to rehabilitation is essential. Guidance from the American Association of Cardiovascular and Pulmonary Rehabilitation (AACVPR) stratifies patients into low, intermediate, and high-risk categories based on parameters such as left ventricular ejection fraction, arrhythmic burden, ischemia, and functional capacity, with exercise type, intensity, and progression individualized accordingly [41].

Moderate-intensity continuous training, typically prescribed at 40–70% of heart rate reserve or peak oxygen uptake, remains the preferred initial modality after PCI. In more deconditioned patients, shorter sessions may be used initially, with progression first in duration and then in intensity. Resistance training is generally introduced after initial aerobic conditioning, with timing individualized according to clinical stability, functional status, access-site healing, ischemia, and arrhythmic risk.

Long-term exercise volume should follow established cardiovascular prevention recommendations, as discussed above. However, no specific exercise intensity, duration, or cumulative dose has been validated for the prevention of ISR. Exercise prescription should therefore be guided by overall cardiovascular benefit, safety, functional capacity, and the likelihood of sustained adherence rather than by a presumed anti-restenotic threshold.

### Safety of Early Exercise after PCI

Available contemporary evidence indicates that early structured exercise in clinically stable patients is associated with low rates of adverse events. In a large French prospective multicenter cohort of 3,132 patients undergoing cardiac rehabilitation after coronary stenting (86.4% for an acute coronary syndrome, across 44 centers), supervised exercise training was associated with a very low incidence of exercise-related cardiovascular events and stent thrombosis, occurring both early and late after PCI [42]. Observational data on previously active individuals resuming higher-intensity exercise within the first year after PCI have likewise shown no excess cardiovascular risk compared with lower-intensity activity, with rare exercise-triggered events overall [43]. Randomized trials of supervised high-intensity interval training after PCI reinforce this safety signal, showing no increase in major adverse cardiovascular events together with reductions in inflammatory biomarkers implicated in restenosis [33]. A prospective observational study of 3,672 patients with stable coronary artery disease similarly found comparable rates of stent thrombosis and major adverse cardiovascular events between exercise-trained and control patients, with fewer unscheduled hospital visits for angina among exercise participants [44].

Collectively, these studies do not indicate an excess risk associated with appropriately prescribed exercise after PCI. Nevertheless, serious complications such as stent thrombosis are uncommon, and most available studies were not powered to exclude small differences in rare events. Moreover, participants undergoing high-intensity training were generally selected and closely supervised. The evidence therefore supports early, individualized exercise in clinically stable patients, while caution remains appropriate when extrapolating these findings to unsupervised training or higher-risk populations.

### Exercise Intensity and Clinical Monitoring

Among the cornerstone strategies of secondary prevention after acute coronary syndromes, structured physical exercise plays a pivotal role, with well-established benefits on cardiovascular outcomes and functional capacity; however, key aspects such as optimal intensity, modality, and monitoring remain incompletely defined and likely require a patient-tailored, phenotype-oriented approach.

Robust observational evidence supports the prognostic benefit of cardiac rehabilitation. In a cohort of 2,986 patients undergoing rehabilitation after PCI, participation was associated with a 33% reduction in long-term mortality at 6 years; outcomes were strongly influenced by adherence, with completion of at least 36 exercise sessions associated with up to a 50% lower risk of death [45]. In this context, increasing attention has been directed toward high-intensity interval training (HIIT), given its potential to induce superior cardiovascular adaptations, although its optimal patient selection remains under active investigation. Emerging evidence suggests that HIIT-based rehabilitation after PCI may be associated with lower levels of systemic inflammation, reflected in reduced interleukin-6 and C-reactive protein, paralleled by less late luminal loss, a pattern that plausibly contributes to the vascular benefits attributed to more intensive training regimens [46].

Cardiorespiratory fitness, typically assessed by cardiopulmonary exercise testing and expressed as peak oxygen uptake (VO2peak), is a key marker of the integrated cardiac, pulmonary, and skeletal muscle adaptation to training. A meta-analysis of 22 clinical trials (*n* = 949) demonstrated the superiority of HIIT over moderate-intensity continuous training in improving cardiorespiratory fitness, with comparable safety profiles; the greatest improvements in VO2peak were observed in patients completing at least three HIIT sessions per week for a minimum of 12 weeks [47]. These findings should nonetheless be interpreted with caution: high-intensity exercise is not universally applicable and may not suit all patient subsets, particularly those at higher clinical risk, and the closely supervised settings in which HIIT is typically delivered, with intensive clinical monitoring, may themselves contribute to the low rate of observed adverse events, a level of supervision that can be difficult to replicate in routine practice due to logistical and economic constraints, limiting the generalizability of these findings [47].

Although definitive evidence on optimal exercise intensity is still lacking, current guideline recommendations emphasize early initiation of exercise-based cardiac rehabilitation for patients undergoing coronary artery bypass grafting, cardiac surgery, or PCI, typically within a structured program lasting 8–12 weeks after hospital discharge; delays in initiation may attenuate benefit, with each week of postponement potentially requiring a substantially longer duration of training to achieve comparable clinical improvement [38]. Irrespective of exercise intensity, a structured, cardiology-led evaluation remains essential both at baseline and throughout the rehabilitation pathway, systematically including electrocardiographic assessment, cardiovascular imaging, frailty evaluation, and objective measurement of peak exercise capacity, an integrated approach that is crucial not only to individualize exercise prescription, but also to ensure safety, enable early detection of clinical instability, and optimize long-term outcomes.

### Clinical Implications and Future Directions

Direct evidence on the impact of physical exercise on vascular healing after PCI remains limited, in part because assessing the stented segment serially requires invasive optical coherence tomography or intravascular ultrasound, or lower-resolution cardiac computed tomography, and in part because a favorable post-PCI clinical trajectory (fewer cardiovascular events, improved functional capacity) is often taken, in routine practice, as an indirect marker of adequate stent re-endothelialization, so that further imaging evaluation is not systematically pursued. Studies of vascular healing response have been conducted with different stent platforms [48–50], but the association between this healing response and physical activity has not yet been systematically investigated with contemporary devices.

Taken together, current findings suggest that aerobic exercise may promote vascular healing after PCI by reducing inflammation, improving endothelial function, and enhancing vasodilation, with the potential, still incompletely proven, to lower the long-term risk of restenosis and major adverse cardiovascular events.

Further prospective studies, ideally incorporating standardized intracoronary imaging endpoints, are needed to clarify the relationship between exercise intensity, duration, and vascular healing after stent implantation, and to determine whether selected high-risk subgroups (complex anatomy, long stented segments, multiple stents, diabetes) derive a disproportionate anti-restenotic benefit from structured training.

Until such data are available, the totality of evidence supports early, individualized, and supervised exercise-based cardiac rehabilitation as safe and beneficial after coronary stenting, justified by its established effects on functional capacity and cardiovascular prognosis, and biologically coherent with, even if not yet proven to directly prevent, in-stent restenosis.

## Conclusion

Exercise training acts on several of the same biological pathways, endothelial dysfunction, inflammation, and neointimal proliferation, that underlie in-stent restenosis, providing a coherent mechanistic rationale for its use as an adjunctive strategy after coronary stenting. Clinical trial and meta-analytic data lend some support to a direct anti-restenotic effect, but the evidence base is dominated by small, heterogeneous studies conducted largely in the bare-metal and early-generation DES era, so a specific benefit on ISR in the contemporary thin-strut DES landscape cannot yet be considered established. More firmly established is the broader safety and clinical value of exercise-based cardiac rehabilitation: available contemporary data indicate that, in clinically stable patients, early, individualized, and supervised exercise after PCI is well tolerated and does not appear to increase the risk of stent thrombosis, clinically relevant arrhythmias, or adverse ventricular remodeling. These findings support prompt initiation of cardiac rehabilitation as a time-sensitive component of post-PCI care, irrespective of its still-unproven direct effect on restenosis. Larger, contemporary randomized trials with standardized imaging endpoints, adequately powered for angiographic and clinical outcomes and stratified by ISR risk profile, are warranted to determine whether, and in which patients, exercise training can be considered a genuine anti-restenotic intervention rather than solely a driver of the broader, already well-established benefits of cardiac rehabilitation.

## Key References

- Li T, Jiang H, Ding J. The role of exercise-based cardiac rehabilitation after percutaneous coronary intervention in patients with coronary artery disease: a meta-analysis of randomised controlled trials. Acta Cardiol. 2024;79(2):127–135. 10.1080/00015385.2023.2266650.
  - ○ A recent meta-analysis directly evaluating exercise-based cardiac rehabilitation after PCI. It reported a favorable signal for coronary restenosis, while also highlighting that the restenosis estimate was based on a limited number of contributing trials.
- Zhang P, Niu C, Zhang L, Lai H, Liu B, Lv D, et al. The impact of the time factors on the exercise-based cardiac rehabilitation outcomes of the patients with acute myocardial infarction after percutaneous coronary intervention: a systematic review and meta-analysis. BMC Cardiovasc Disord. 2024;24(1):35. 10.1186/s12872-023-03692-z.
  - ○ A contemporary systematic review and meta-analysis addressing the initiation and duration of exercise-based rehabilitation after PCI for acute myocardial infarction, with particular relevance to the safety and timing of post-PCI exercise.
- Spadafora L, Quarta R, Martino G, Romano L, Greco F, Curcio A, et al. From mechanisms to management: tackling in-stent restenosis in the drug-eluting stent era. Curr Cardiol Rep. 2025;27(1):53. 10.1007/s11886-025-02193-z.
  - ○ A contemporary review integrating the incidence, biological mechanisms, intracoronary imaging characterization, and mechanism-based management of ISR in the modern drug-eluting stent era.
- Liu B, Li M, Liu J, Xie L, Li J, Liu Y, et al. Risk factors and incidence for in-stent restenosis with drug-eluting stent: a systematic review and meta-analysis. Rev Cardiovasc Med. 2024;25(12):458. 10.31083/j.rcm2512458.
  - ○ A contemporary systematic review and meta-analysis quantifying the incidence of DES-ISR and identifying major patient-, lesion-, and procedure-related risk factors.
- Dibben GO, Faulkner J, Oldridge N, Rees K, Thompson DR, Zwisler AD, Taylor RS. Exercise-based cardiac rehabilitation for coronary heart disease: a meta-analysis. Eur Heart J. 2023;44(6):452–469. 10.1093/eurheartj/ehac747.
  - ○ A large contemporary meta-analysis establishing the broader clinical, prognostic, and quality-of-life benefits of exercise-based cardiac rehabilitation in coronary heart disease.

## Data Availability

No datasets were generated or analysed during the current study.
